# *Babesia microti* Protein *Bm*SP44 Is a Novel Protective Antigen in a Mouse Model of Babesiosis

**DOI:** 10.3389/fimmu.2020.01437

**Published:** 2020-07-07

**Authors:** Hui Wang, Yao Wang, Jilei Huang, Bin Xu, Junhu Chen, Jianfeng Dai, Xia Zhou

**Affiliations:** ^1^School of Biology and Medical Science, Soochow University Medical College, Suzhou, China; ^2^Key Laboratory of Parasite and Vector Biology of the Chinese Ministry of Health, Chinese Center for Disease Control and Prevention, WHO Collaborating Center for Tropical Diseases, National Center for International Research on Tropical Diseases, National Institute of Parasitic Diseases, Shanghai, China; ^3^Jiangsu Key Laboratory of Infection and Immunity, Institutes of Biology and Medical Sciences, Soochow University, Suzhou, China

**Keywords:** *Babesia microti*, secreted protein, vaccine, antigens, diagnosis marker

## Abstract

Babesiosis caused by *Babesia* species imposes an increasing threat to public-health and so far, there is no effective vaccine to prevent *Babesia* infections. *Babesia* surface antigen may participate in the invasion of erythrocytes. In our previous study, a surface antigen of *B. microti* merozoites, named as *Bm*SP44 was identified as a dominant reactive antigen by protein microarray screening. To evaluate its potential applications in diagnosis and prevention of Babesiosis, the open reading frame encoding *Bm*SP44 was cloned and the recombinant protein was expressed. In consistent with the protein microarray result, recombinant *Bm*SP44 (r*Bm*SP44) can be recognized by sera from *B. microti* infected mice. Immunofluorescence assays (IFA) confirmed that *Bm*SP44 is a secreted protein and localized principally in the cytoplasm of the parasites. The parasitemia and *Babesia* gene copies were lower in mice administered r*Bm*SP44 antisera compared with normal controls. Active immunization with r*Bm*SP44 also afforded protection against *B. microti* infection. The concentrations of hemoglobin in r*Bm*SP44 immunization group were higher than those in the control group. Importantly, vaccination of mice with r*Bm*SP44 resulted in a Th1/Th2 mixed immune response with significantly elevated IL-10 and IFN-γ levels during the early stage of infection. Taken together, our results indicated that r*Bm*SP44 can induce a protective immune response against *Babesia* infection. Thus, *Bm*SP44 can be used as both a diagnosis marker and a vaccine candidate.

## Introduction

*Babesia* is a tick-borne intraerythrocytic protozoan parasite belonging to the phylum Apicomplexa. *B. microti* causes babesiosis in animals and humans worldwide. Although most human babesiosis cases were reported in the United States (1), babesiosis is regarded as an emerging vector-borne parasitic disease in other countries during recent years (2, 3). Babesiosis is normally a benign infection and most of the cases can be asymptomatic or present with mild symptoms. But in populations of neonates or immunocompromised patients, the infection of *Babesia* can be fatal (4, 5). Currently, no vaccine is available to control *Babesia* infection, and drugs for babesiosis are limited, suggesting the importance and necessity to explore potential vaccine based on relative antigen molecules (6, 7).

In recent years, several proteins involved in cell invasion and immunity have been developed as vaccine candidates and their protections against *Babesia* infection in animal models have been evaluated (8–13). But all these candidates as vaccines exhibited limited protection from the infection of *Babesia*. Thus, more work is required for identification of novel targets which can induce stronger protection against babesiosis.

The process of parasite invasion and enveloping within host cells is highly dependent on the interaction between the parasite and host-surface molecules (14, 15). The surface proteins produced by *Babesia* parasite enable it to adhere to and invade the erythrocytes, where it survives and grows (16, 17). Surface secreted proteins usually play a key role in facilitating parasite invasion, host cell remodeling and can be targeted or activated by the humoral immune response in the host (18–21). The surface proteins present in early infective stages may be useful for developing a diagnostic test for babesiosis as well as vaccine.

In our previous study, protein microarray screening was performed using a *Babesia* genomic expression library against murine sera from different stages of infection. Ten *B. microti* antigens were identified as targets of host humoral immune responses (22). However, the antigenicity, immunogenicity, function, and subcellular localization of these surface antigens are not clearly understood.

In the current study, the functions of a dominant *Babesi*a antigen *Bm*SP44 was evaluated in a mouse model of babesiosis. *Bm*SP44 was confirmed as a secretory protein in the parasite of *B. microti*. The protection effect of this antigen against *Babesia* infection in a mouse model was examined by passive and active immunization strategies. Meanwhile, the changes of cytokine expressions after active immunization were examined to systematically evaluate the function of the protein. Our results suggested that *Bm*SP44 may serve as a potential vaccine candidate as well as a diagnostic antigen.

## Materials and Methods

### Ethics Statement

All animal procedures were conducted in compliance with the principle for the Care and Use of Medical Laboratory Animals (Ministry of Health, People's Republic of China) and approved by the Institutional Animal Care and Use Committee (IACUC) of Soochow University for the use of laboratory animals (Permit Number: ECSU-201800091). All efforts were made to minimize suffering.

### Animals and *Babesia* Infections

Six-to-eight-week-old female BALB/c mice were provided by the Experimental Animal Center of Soochow University (Suzhou, China), and kept under specific pathogen-free conditions. The Peabody strain (ATCC, PRA-99) of *B. microti* was obtained from ATCC. One mouse was initially infected via intraperitoneal injection and blood from the mouse (5 days post infection, the parasitemia is approximately 60%) was taken from the eyelids, anticoagulated with ethylenediaminetetraacetic acid (EDTA), mixed with sterile 0.9% physiological saline in a ratio of 1:2, and infected by 100 μl per BALB/c mouse via intraperitoneal injection. The infections were performed with this strain of *Babesia* by intraperitoneal (i.p.) injection with 1 × 10^7^ *B. microti*-infected red blood cells (iRBCs). Totally, 6 mice equally divided into two groups, 3 mice in the immunized group, and 3 ones set as the control were use in the passive experiments. And there were totally 10 mice in the active immunized experiment, each group with 5 mice and the same protocols were applied in independently.

### Preparing Secreted Protein Microarray and Acquiring Immunoreactivity Profiles

Based on the SignalP software (http://www.cbs.dtu.dk/services/SignalP/) and EuPathDB database (http://piroplasmadb.org/piro/), ORFs encoding *Babesia* proteins containing signal peptides were selected and cloned in-frame into the pEU-E01-His-TEVMCS-N2 (pEU, Cell Free Sciences, Matsuyama, Japan) vector. The cell-free protein synthesis system, wheat germ cell-free (WGCF) system was applied in the high throughput expression assay. The ORF sequences were amplified from cDNA of *B. microti* PRA99 strain and the recombinant protein r*Bm*SA1 (23) expressed in *E. coli* DH5α strain was used as the positive control. The wheat germ lysate with expression of an empty vector served as the negative control. Detailed protocols with the high throughput assay were described in our previously study (22).

### Bioinformatic Analysis of the Gene Coding for *Bm*Sp44

Antigenic epitopes were predicted using ABCPred (http://www.imtech.res.in/raghava/abcpred/). The hydrophobicity and the signal peptide were predicted using Expasy (http://www.expasy.org/) and the SignalP4.1 server (http://www.cbs.dtu.dk/services/SignalP/), respectively.

### Expression and Purification of Recombinant Protein *Bm*SP44

Expression of *Bm*SP44 with glutathione S-transferase (GST) fusion proteins was conducted using the pGEX vector system. ORF of *Bm*SP44 was amplified with the proof-reading Polymerase (Pfu) (Transgene, Beijing) from the cDNA of *B. microti* using gene-specific primers (forward-TTCCAGGGGCCCCTGGGATCCATGCATATCAACTACAAATTAATTA and reverse- TCACGATGCGGCCGCTCGAGTTAAGCAGCATTAGGTGTGTGAT). The fragment was then cloned into pGEX-6P-2 (Invitrogen, Carlsbad, NM) vector by digestion with *BamHI* and *XhoI* (Vazyme, USA). Validated pGEX constructs were re-transfected into *E. coli* strain BL21 (DE3) for recombinant protein expression. Briefly, 1,000 ml of LB medium containing 1 ml of ampicillin were incubated with bacterium in constant temperature shaker. After 4 h, protein expression was induced with 0.5 mM Isopropyl β-D-1-thiogalactopyranoside (IPTG). Then, the soluble recombinant GST-tagged fusion proteins were purified using GST affinity agarose (GE Healthcare, Sweden) and the GST tag was removed by on-column enzyme digestion of Prescission Protease (Sigma).

### Preparing Rabbit Antisera Against r*Bm*SP44

For generation of Rabbit antisera against r*Bm*SP44, NZW rabbit was immunized with 100 μg of r*Bm*SP44 together with complete Freund's adjuvant (Sigma-aldrich, USA). The rabbit was received 2 boosts (400 μg r*Bm*SP44) at 2-week intervals in incomplete Freund's adjuvant. Two weeks after the final boost, sera were collected and the antibody titers were evaluated with the standard ELISA procedures described above.

### Western Blot Analysis

Total protein samples were separated on 10% sodium dodecyl sulfate polyacrylamide gel electrophoresis (SDS-PAGE), and transferred to a 0.45 μm polyvinylidene difluoride (PVDF) membranes. The blot was blocked with 5% skim milk diluted in TBS containing 0.05% Tween (TBST) for 1 h at room temperature. The *B. microti* hyperimmune sera were diluted (1:5,000) in TBST containing 2% skim milk and incubated overnight at 4°C. The blot was washed with TBST three times and then incubated in an HRP-conjugated goat anti-mouse IgG (H+L) secondary antibody (Bioworld, USA) (1:10,000) for 1 h at room temperature. After three washes with TBST, the signal was detected with an enhanced chemiluminescence ECL Plus kit (Thermo, USA).

### Evaluation of r*Bm*SP44 as a Diagnostic Antigen

Enzyme-linked immunosorbent assay (ELISA) plates were coated with the r*Bm*SP44 protein (to the final concentration of 5 μg/ml) and incubated overnight at 4°C. After three washes with PBST, the plates were blocked with 2% BSA. One hundred microliters of sera from different infection stages (7, 14, and 21 days post infection), and negative mouse sera (from healthy mice) were diluted (1:2,000) in 2% BSA and incubated for 30 min. After incubating with peroxidase-conjugated rabbit anti-mouse IgG antibody, the reaction was examined with 3, 3, 5, 5′-Tetramethylbenzidine–hydrogen peroxide substrate (TMB) (Biolegend, USA) according to standard protocols.

### Immunofluorescent Assay (IFA) and Confocal Microscopy

Anticoagulated blood collected from mice infected with *B. microti* with approximately 30% parasitemia was smeared on slides using cytospin centrifugation (Thermo Fisher Scientific) and fixed with 4% PFA-PBS for 10 min. After washed three times with PBS, the slides were permeabilized with 0.4% Triton X100 for 10 min, and then treated with protease K (20 μg/ml) for 5 min. After washes, the slides were blocked with 5% FBS for 30 min to reduce non-specific binding and then incubated overnight at 4°C with a 1:300 dilution of mouse anti-BmSP44 serum. After washing three times again, the slides were incubated with Alexa Fluor488 goat anti-mouse IgG for 1 h (Invitrogen) diluted at 1:500 in PBS for 1 h at room temperature. After washes, the slides were incubated with 0.5 μg/ml 6-diamidino-2-phenylindole (DAPI) for 15 min and imaged using a Nikon C2 Confocal microscope system (Nikon, Tokyo, Japan).

### Passive Immunization

The rabbit was immunized with r*Bm*SP44 100 μg mixed with complete Freund's adjuvant (Sigma-aldrich, USA) in ratio of 2:1 and boosted twice at 2-week intervals by injection of 200 μg protein mixed with incomplete Freund's adjuvant also in ratio of 2:1. Two weeks after the final boost, sera were collected and the antibody titers were evaluated with the standard ELISA procedures described above. For passive immunization, mice were administered of r*Bm*SP44 rabbit antisera (200 μl each) or control antisera (5 per group). Twenty-four hours later, the animals were challenged with intraperitoneal inoculation of 1 × 10^7^ *B. microti*-parasitized red blood cells (iRBCs).

### Active Immunization

For active immunization, mice (5 per group) were immunized with r*Bm*SP44 (20 μg/each) mixed with complete Freund's adjuvant while the control group receiving Freund's adjuvant only. Animals were received 2 boosts (40 μg r*Bm*SP44) at 2-week intervals in incomplete Freund's adjuvant. Two weeks after the final boost, sera were collected and evaluated with the standard ELISA procedures described above.

### Parasitemia and *Babesia* Load

The concentration of parasites in the blood was determined by blood smears examination and quantitative RT-PCR (qRT-PCR). Thin films from the peripheral blood were prepared every third days after *B. microti* inoculation, and stained with Giemsa's solution. The numbers of infected and non-infected erythrocytes were counted per 50 microscopic fields to calculate parasitemia. The degree of infection in each group is presented as the geometric mean of the parasitemia percentage. The blood RNA was extracted used for using the blood RNA kit (OMEGA, USA). Briefly, the blood RNA was reverse-transcribed using the PrimeScript Master Mix kit (TaKaRa, Japan). PCR was performed by using iTaq SYBR Green Supermix (Monad, China) on a CFX96 real time PCR system (Eppendorf, USA) and involved an initial denaturation at 95°C for 30 s, 40 cycles of 5 s at 95°C, and 30 s at 60°C. At the end of each reaction, a melting curve (70–95°C) was checked to confirm the identity of the PCR product. *B. microti 18S rRNA* primers: forward- AGCGTTTTCGAAGGTATGTTGC and reverse-AGCAGATACATCCTTACTAGGGAAA. Mouse GAPDH (control): forward- GGCCTTCCGTGTTCCTACC and reverse – AGCCCAAGATGCCCTTCAGT were applied during the amplification. And the mouse GAPDH gene was amplified as an internal control.

### Severity of the Disease

Besides the parasitemia and *Babesia* load, the weight, temperature, and hemoglobin level of mice were also measured to evaluate the severity of the disease. The weight and the temperature were monitored daily post *B. microti* infection. To measure the hemoglobin (Hb) concentration, 10 μl of blood was diluted in 2,490 μl of Drabkin's reagent (Sigma-Aldrich, St. Louis, MO, USA) and quantified at 540 nm using a biophotometer (Eppendorf, USA). The absorbance and hemoglobin concentration (Hb) were counted using a commercial Hb standard curve.

### Detection of Cytokine Levels in Serum

After receiving active immunization with the recombinant protein, mice were challenge with 1 × 10^7^ *B. microti* infected erythrocytes. Sera samples were prepared from each immunized or control mouse on day 0, 3, 6, and 9 after challenge. The concentration of cytokines such as IFN-γ, TNF-α, and IL-10 were determined by ELISA assay according to the manufacturer's instructions (Biolegend, USA).

### Statistical Analysis

GraphPad Software of Prism 7 software was used in charts and statistical analyses. Statistical differences were analyzed by Student's *t*-test and ANOVA. A value of *p* < 0.05 was considered statistically significant.

## Results

### High-Throughput Screening of Secreted Proteins by Protein Arrays

**Figure 1:**
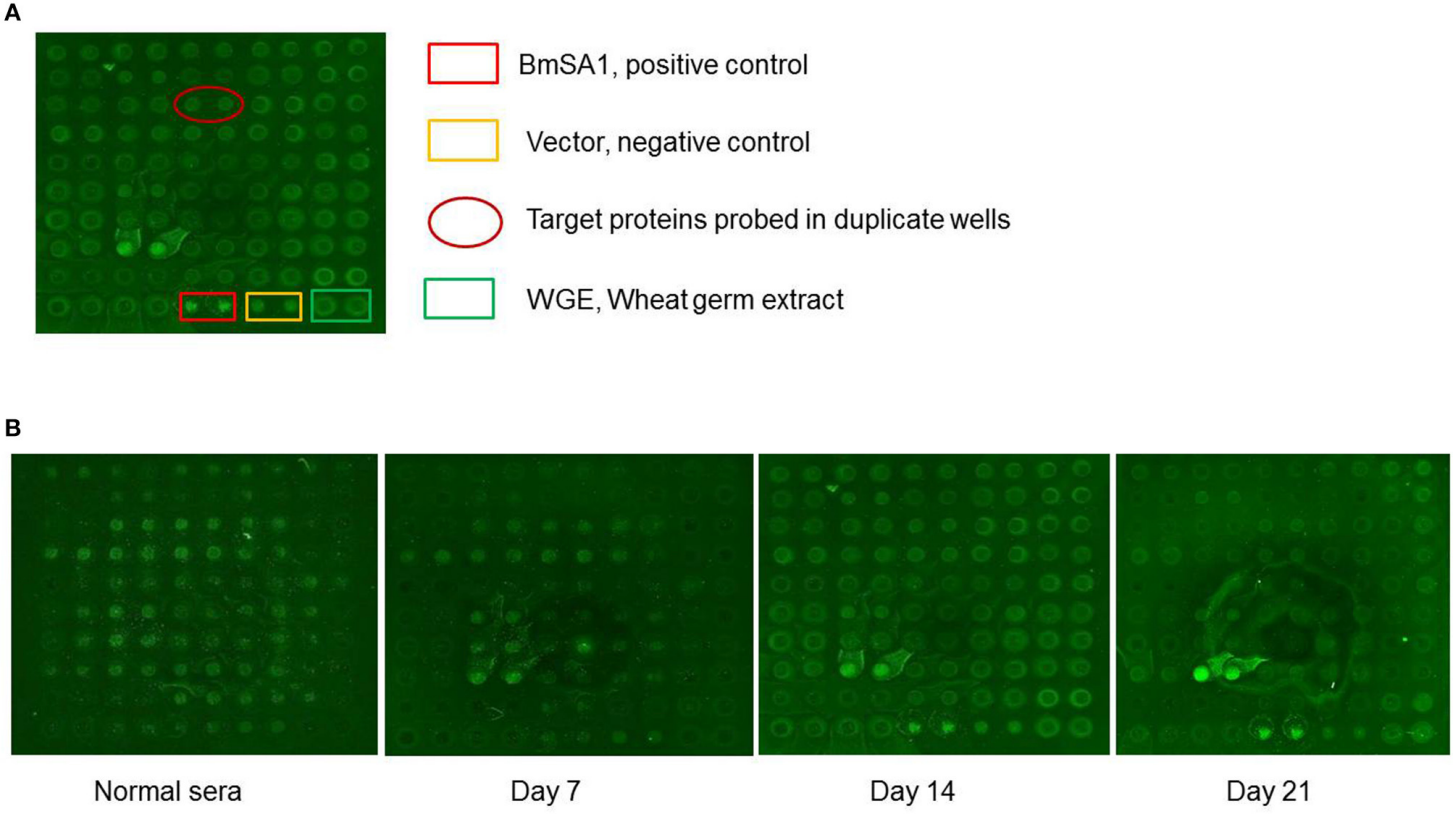
*B. microti* proteins detected by sera from infected BALB/c mice. **(A)** Normal: the sera from the normal BALB/c mouse; the sera from the BALB/c mice infected on day 7, 14, and 21 p.i. pEU-His vector, Wheat germ extract (WGE) and *Bm*SA1 were used as negative and positive controls, respectively. **(B)** The protein arrays probed with mouse sera from 3 different infection stages.

A total of 55 proteins containing signal peptides from *B. microti* were selected and screened by sera collected from *B. microti* infected mice at different stages of infection. One secreted protein, named *Bm*SP44, showed a higher immune reactivity with the sera collected on day 7, 14, and 21 post infection compared to other antigens tested (Figures 1A,B).

### Molecular Characterization and Sequence Analysis of *Bm*SP44

The nucleotide sequence coding *Bm*SP44 was 654 bp long and predicted to encode a protein of 218 amino acid residues (Supplementary File 1 with a predicted molecular weight and isoelectric point of 24 kDa and 5.39. Signal peptide sequence was found in *Bm*SP44 protein based on SignalP4.1 and antigenic epitopes were predicted using ABCPred bioinformatic serves (Supplementary Figures 1A,B. However, no homologous genes were identified in other species of *Babesia* based on the current genome sequences available from NCBI database.

### Evaluation of *Bm*SP44 as a Diagnostic Antigen

**Figure 2:**
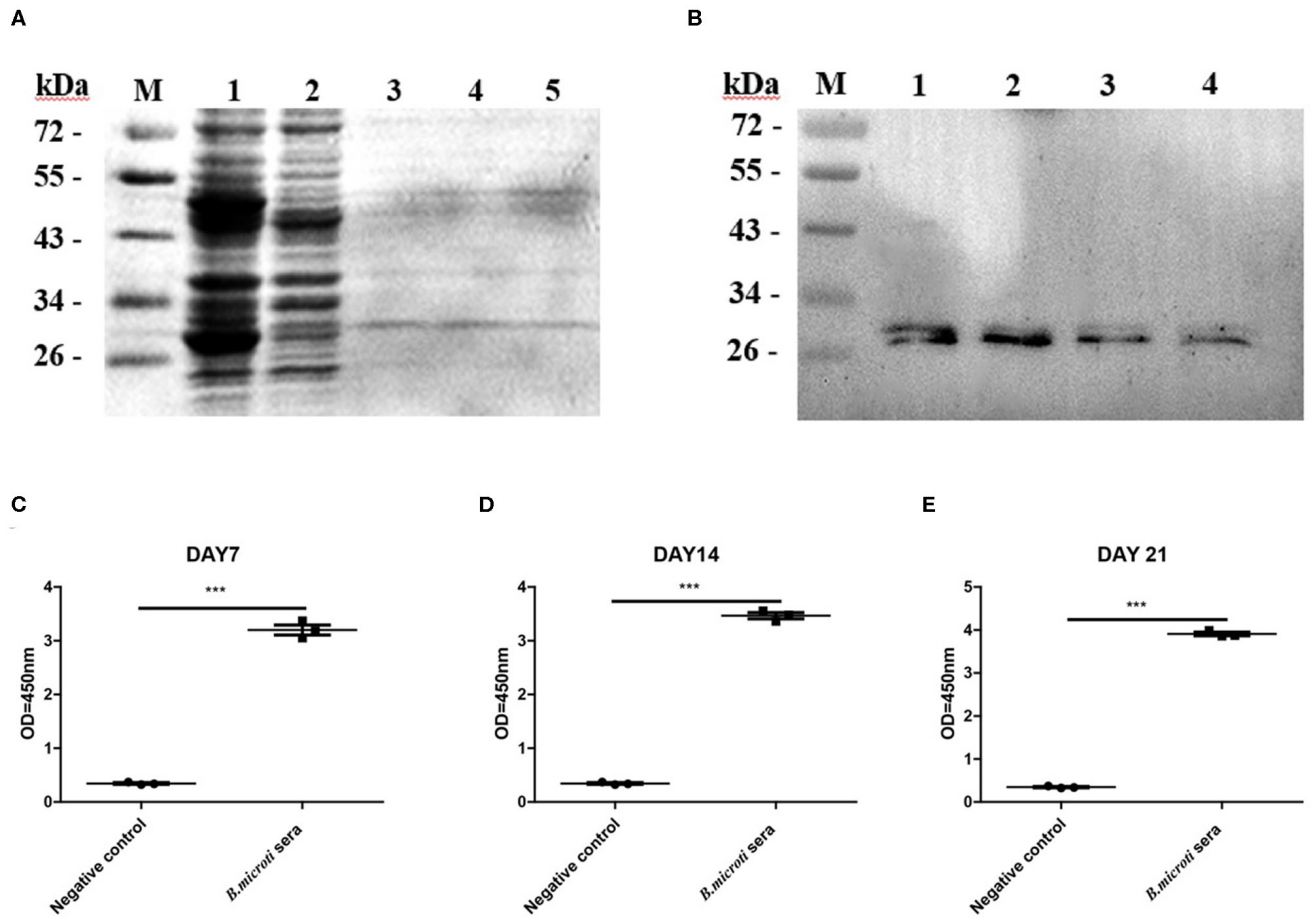
Recombinant antigen production and evaluation of r*Bm*SP44 as a diagnostic antigen. **(A)** SDS-PAGE analysis of bacterial lysate as stained by Coomassie blue. Lane 1: induced protein; Lane 2: non-induced control; Lane 3 and 5: The purification of r*Bm*SP44 after cleavage of GST tag; **(B)** Western blot analysis of r*Bm*SP44; M: Protein marker; Lane 1–4: The recombinant proteins; Protein incubated with an anti-r*Bm*SP44 monoclonal antibody (~24 kDa); **(C–E)** r*Bm*SP44 recognized by sera from *B. microti* infected mice [Sera from day7 **(C)**, 14 **(D)**, and 21 **(E)** p.i.] Significant differences are as follows: ***P < 0.001, compared to non-infected control sera, *t*-test. The data shown are representative of at least 3 independent experiments.

To investigate the characteristics of *Bm*SP44, a recombinant protein without the GST tag (~24 kDa) was produced in the *E.coli* expression system (Figure 2A) and used to immunize mice. Western blot suggested that r*Bm*SP44 reacted with the sera from r*Bm*SP44-immunized mice but not from the controls (Figure 2B).

To validate the potential of r*Bm*SP44 as a diagnostic antigen, an indirect ELISA was set to detect *Bm*SP44 specific antibodies from sera of *B. microti* infected mice. The results suggested that r*Bm*SP44 can be detected with mouse serum collected on day 7, 14, and 21 p.i. (Figures 2C–E), which were consistent with the data from high throughput protein microarray. Thus, *Bm*SP44 can be used as a diagnostic antigen since it can be detected with host serum at different stages of infection.

### Localization of *Bm*SP44 in *B. microti*

**Figure 3:**
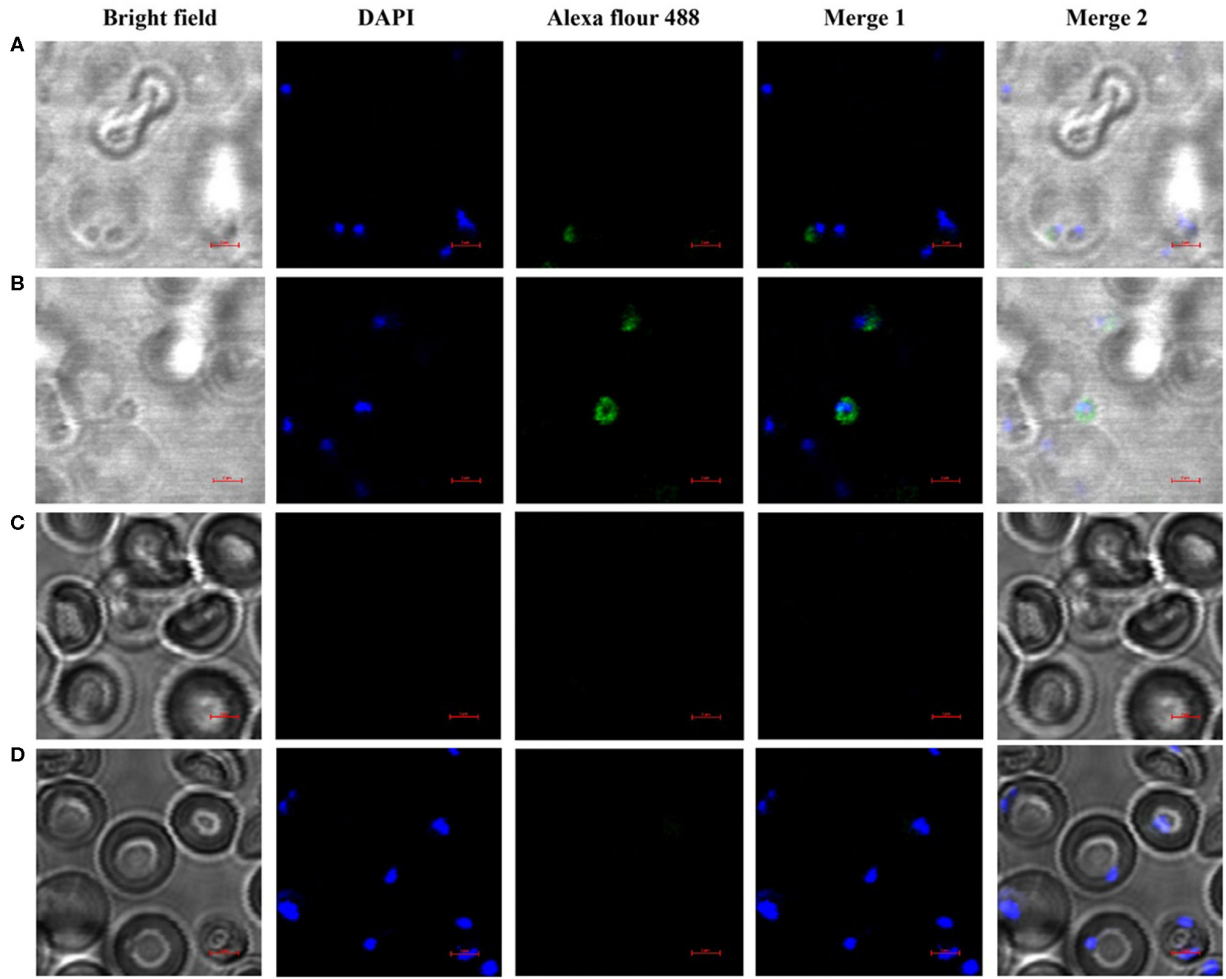
*Bm*SP44 is a secreted protein pf *B. microti*. The immunolocalization of *Bm*SP44 on the membrane of the *B. microti* and the cytoplasm of the iRBCs were examined by IFA with anti-*Bm*SP44 sera. The obvious green fluorescent staining is observed on the membrane of the parasite and the cytoplasm of the iRBCs. **(A)** iRBCs incubated with mouse anti- r*Bm*SP44 serum, the parasite taking residence in RBCs; **(B)** iRBCs incubated with mouse anti- r*Bm*SP44 serum, the parasite outside the RBCs; **(C)** Normal RBCs incubated with mouse anti-r*Bm*SP44 serum; **(D)** iRBCs incubated with normal mouse serum; Bars, 2 μm.

The localization of *Bm*SP44 in *B. microti* and infected RBCs was detected by IFA using mouse anti-*Bm*SP44 sera. *Bm*SP44 appeared to localize on the surface of *B. microti*, supporting that it is a potential secreted antigen of *B. microti* (Figures 3A,B). As negative controls, red blood cells from non-infected mice did not show any specific fluorescent signals (Figures 3C,D).

### Passive Immunization With Antisera Against r*Bm*SP44 Interferes With *B. micoti* Infection

**Figure 4:**
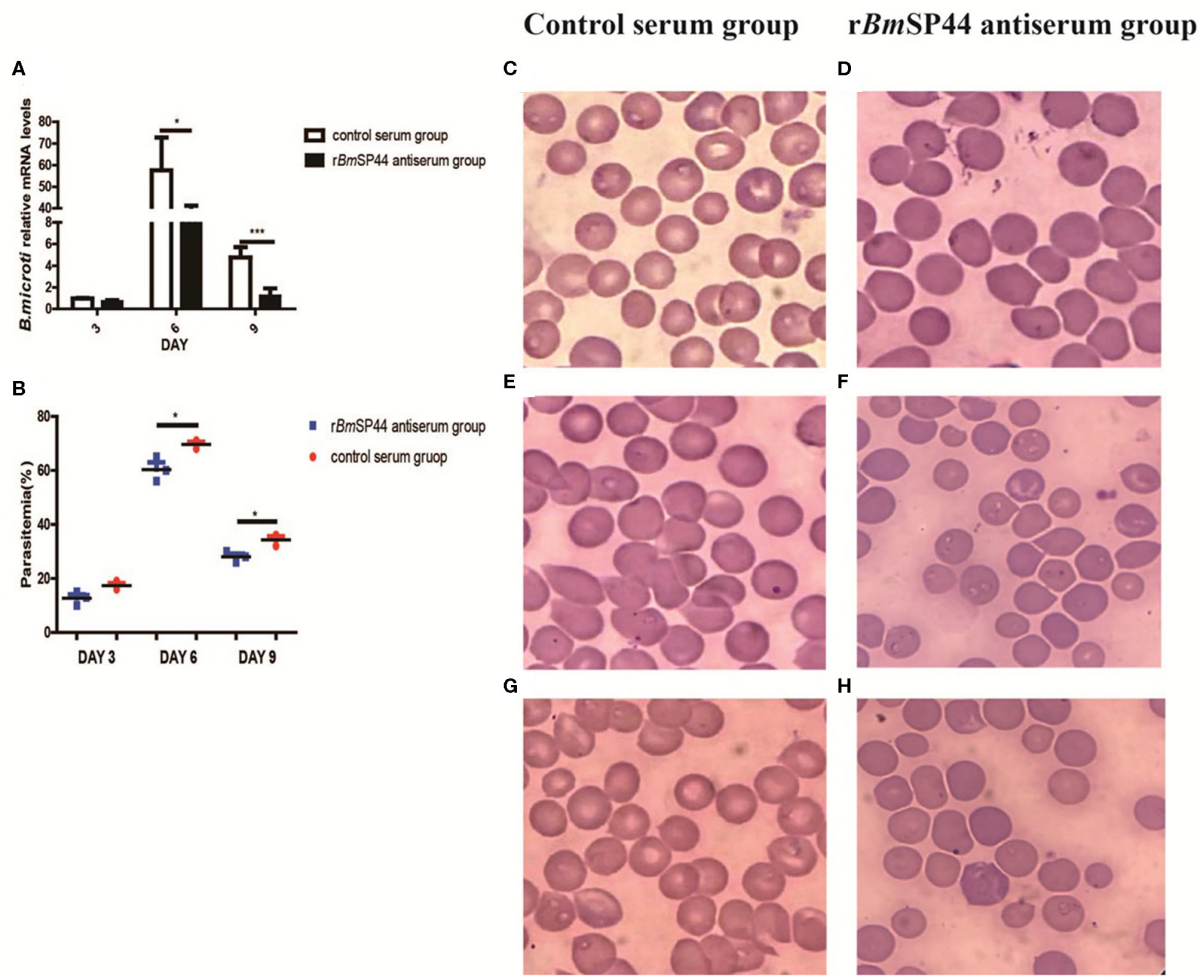
The Parasitemia were reduced in *Bm*SP44 antisera passive immunized mice. **(A)** Revervse transcription qPCR analysis of the expression of *B. microti* at different developmental stages. After reverse transcription, the cDNA was used as a template for qPCR using SYBR Green. Each reaction was performed in triplicate. All fold changes were relative to the different stage. Significant differences are as follows: *P < 0.05, ***P < 0.001. **(B)** Parasitemia of the immunized group is lower than that of the control group, which is quantified as the percentage of iRBCs by blood smears on day 3, 6, and 9 p.i. The degree of parasitemia were 16, 70, and 32% in normal rabbit sera passive immunized group (*n* = 3) and 10, 56, and 18% in r*Bm*SP44-antiserum immunized group (*n* = 3), respectively. The values shown for each group are the mean + SEM of the parasitemia. Significant differences are as follows: *P < 0.05. **(C–H)** Giemsa staining of blood smears from group **(C,E,G)** and r*Bm*SP44-antiserum group **(D,F,H)**. Sections obtained on day 3 **(C,D)**, 6 **(E,F)**, and 9 **(G,H)** p.i.

In order to examine whether r*Bm*SP44 antiserum thwarts infection with *B. microti*, r*Bm*SP44 antisera were administered into the mice 24 h before *B. microti* challenge. The titers of the rabbit antisera against r*Bm*SP44 were confirmed by ELISA before inoculation (Supplementary Figure 2). qRT-PCR analysis suggested that *Babesia* gene copies in mice receiving rabbit antisera against r*Bm*SP44 were reduced on day 3, 6, and 9 p.i. compared with that in control animals (Figure 4A). The results from the blood smears also showed a decrease parasitemia in passive immunization group compared with the control group (Figures 4B–H).

### Active Immunization With r*Bm*SP44 Reduces *Babesia* Infection in Mice

**Figure 5:**
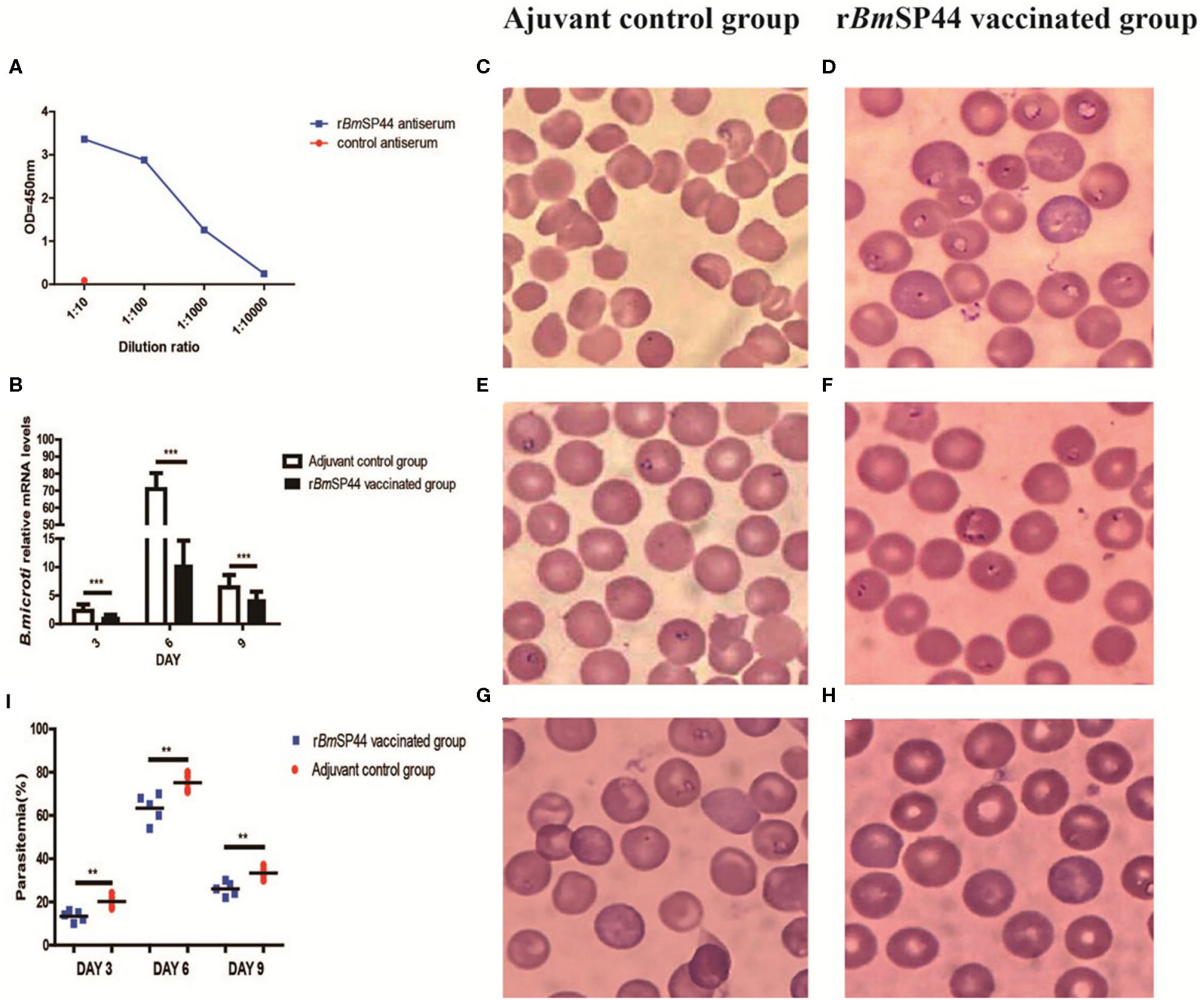
r*Bm*SP44 active immunization protects against *B. microti* infection in BALB/c mice. **(A)** The levels of anti-*Bm*SP44 antibody in the sera of immunized mice or control (adjuvant) mice were measured by ELISA at different time points. **(B)** Active immunization with r*Bm*SP44 or adjuvant control BALB/c mice were challenged with 1 × 10^7^ erythrocytes with *B. microti* and blood samples were obtained on day 3, 6, and 9 p.i. Revervse transcription qPCR analysis of the expression of *B.microti* at different developmental stages. After reverse transcription, the cDNA was used as a template for qPCR using SYBR Green. Each reaction was performed in triplicate. All fold changes were relative to the different stage. Significant differences are as follows: ***P < 0.001. **(C–I)** Parasitaemia is quantified as the percentage of iRBCs by blood smears on day 3, 6, and 9 p.i. Giemsa staining of blood smears from control **(C,E,G)** and r*Bm*SP44 immunized group **(D,F,H)**. Sections obtained on day 3 **(C,D)**, 6 **(E,F)**, and 9 **(G,H)** p.i. **(I)** Parasitemia of the immunized group and that of the control group caculated by blood smears observed under microscope. The degree of infection were 20, 75, and 35% in control Adjuvant-vaccinated group (*n* = 5) and 12, 60, and 24% in r*Bm*SP44 vaccinated group (*n* = 5), respectively. The values shown for each group are the mean + SEM of the parasitemia. Significant differences are as follows: **P < 0.01.

We then examined whether active immunization of mice with r*Bm*SP44 influenced *Babesia* infection. Following active immunization, high levels of r*Bm*SP44 antibodies were detected in mouse sera (Figure 5A). After challenged with *B. microti*, mice immunized with r*Bm*SP44 displayed reduced *Babesia* gene copies in whole blood samples on day 3, 6, and 9 p.i. compared with controls (Figure 5B). A reduced *Babesia* parasitemia also observed in blood smears from r*Bm*SP44 immunized mice compared to controls (Figures 5C–I). Therefore, active immunization with r*Bm*SP44 also protects against *B. microti* infection of mice.

**Figure 6:**
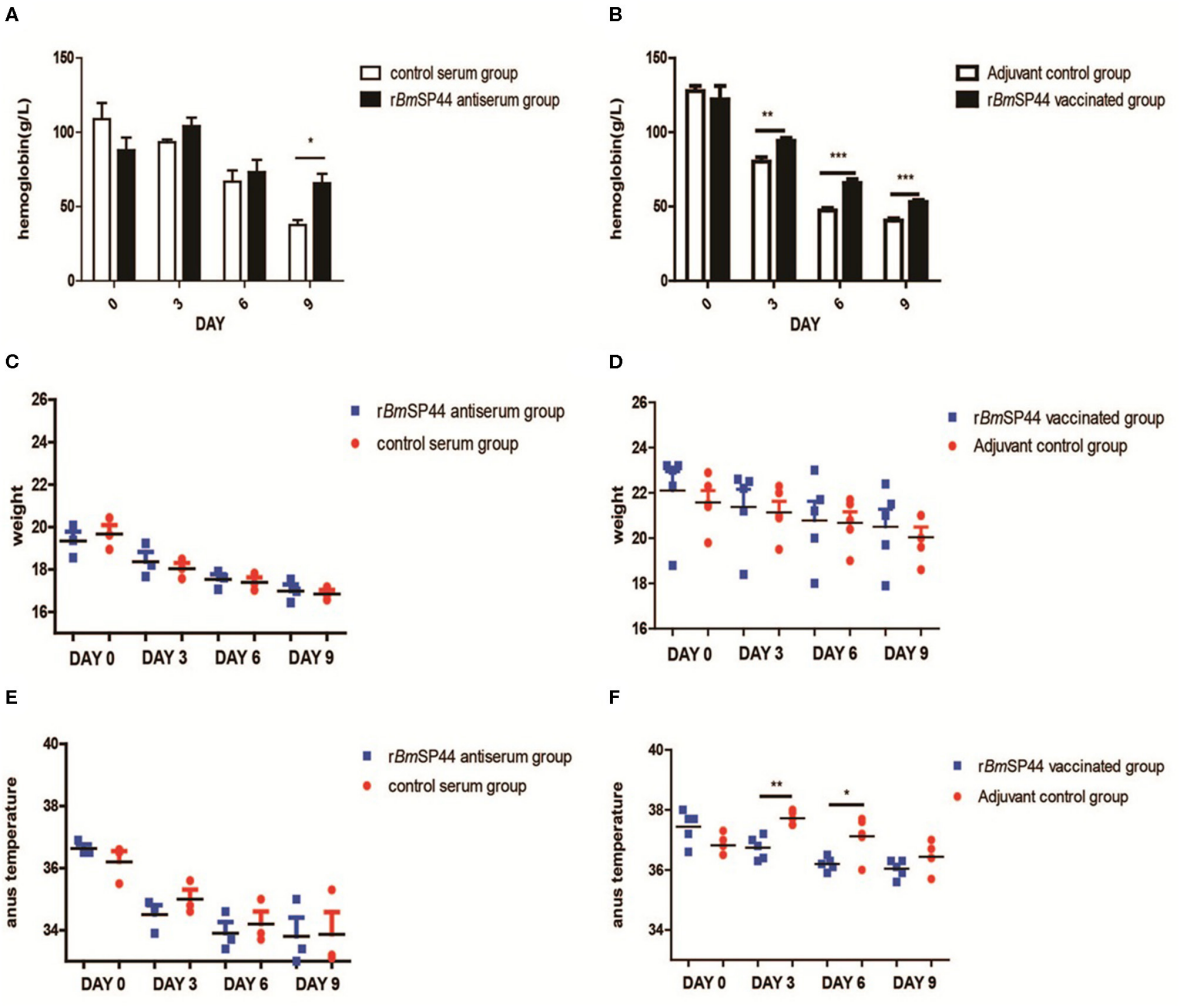
The hemoglobin level, weight, and temperature of *B. microti* infected mice. **(A,B)** The hemoglobin was measured in peripheral blood by Drabkin's reagent on Day 0, 3, 6, and 9 p.i. The concentrations of hemoglobin were 118, 92, 68, and 38 g/l in the normal rabbit sera passive immunized group (*n* = 3); 88, 100, 73, and 72 g/l in r*Bm*SP44-antiserum immunized group (*n* = 3). The data in the active immunized groups are as following: 125, 80, 48, and 41 g/l in control Adjuvant-vaccinated group (*n* = 5) and 118, 92, 63, and 53 g/l in r*Bm*SP44 vaccinated group (*n* = 5), respectively. The values shown for each group are the mean + SEM of the hemoglobin. Significant differences were as follows: *P < 0.05,**P < 0.01, ***P < 0.001. **(C,D)** The change in weight was not discernible on day 0, 3, 6, and 9 p.i. The weight were 19.6, 18.1, 17.3, and 16.8 g in normal rabbit sera passive immunized group (*n* = 3), 19.4, 18.2, 17.6, and 17.0 g in r*Bm*SP44-antiserum immunized group (*n* = 3). The data in the active immunized group are as following: 21.5, 21.0, 20.8, and 20.0 g in control Adjuvant-vaccinated group (*n* = 5) and 23.0, 22.2, 21.2, and 21.0 g in r*Bm*SP44 vaccinated group (*n* = 5), respectively. **(E,F)** The temperatures of the r*Bm*SP44 immunized mice were significantly lower than that of the control mice on day 3 and 6 p.i. The degree of weight were 36.5, 34.8, 33.9, and 33.2^o^C in normal rabbit sera passive immunized group (*n* = 3); 36.5, 34.7, 33.7, and 33.4^o^C in r*Bm*SP44-antiserum immunized group (*n* = 3). The data in the active immunized groups were 36.8, 37.7, 37.2, and 36.4^o^C in control Adjuvant-vaccinated group (*n* = 5) and 37.7, 36.8, 36.2, and 36.1°C in r*Bm*SP44 vaccinated group (*n* = 5), respectively. The starting point of the ordinate in the scatter plot is 33.0^o^C and the values shown for each group are the mean + SEM of the temperature levels. Significant differences were as follows: **P < 0.01, *P < 0.05. The representative results of at least 3 independent experiments are shown, with 3–5 mice per group.

Parasitemia is a major indicator for evaluating the severity of the disease. Meanwhile, weight, body temperature, and concentration of hemoglobin were also introduced to evaluate the severity of Babesiosis. Our results showed that the concentrations of hemoglobin in r*Bm*SP44 immunized mice were significantly higher than that of controls, while there are no obvious differences regarding the weight and the temperature between the two groups (Figures 6A–F).

### Cytokine Profiles of r*Bm*SP44 Immunized Mice

**Figure 7:**
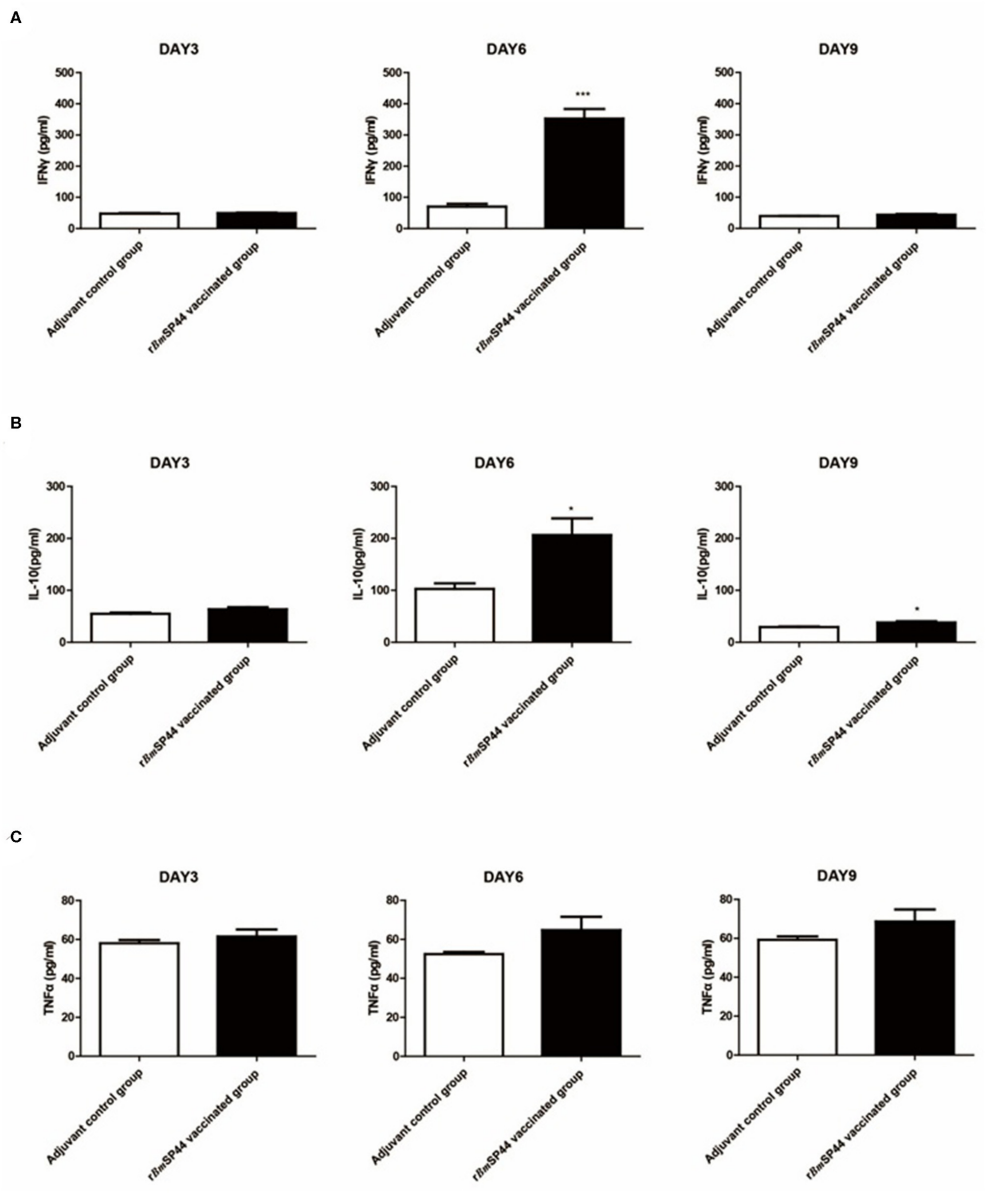
Cytokine levels in sera of r*Bm*SP44 immunized or mice after infection of *B. microti*. BALB/c mice of the *Bm*SP44 vaccinated or adjuvant treated control groups were followed by a challenge with 1 × 10^7^ erythrocytes infected with *B. microti* and the samples were taken on Day 3, 6, and 9 p.i. **(A)** The concentrations of IFN-γ in sera were 45.4, 60.5, and 40.0 pg/ml in Adjuvant-vaccinated control group (*n* = 5) and 52.3, 231.9, and 43.5 pg/ml in r*Bm*SP44 vaccinated group (*n* = 5), respectively. Results are presented as the mean ± SEM. The asterisks (*) indicate that the levels of IFN-γ by immunized mice was significantly higher (***P < 0.001) than those of the adjuvant control group. **(B)** The concentrations of IL-10 in sera were 53.9, 101.5, and 28.8 pg/ml in Adjuvant-vaccinated control group (*n* = 5) and 64.4, 193.2, and 40.9 pg/ml in r*Bm*SP44 vaccinated group (*n* = 5), respectively. Results are presented as the mean ± SEM. The asterisks (*) indicate that the levels of IL-10 by immunized mice were significantly higher (*P < 0.05) than those of the adjuvant control group. **(C)** The concentrations of TNF-α in sera were 57.2, 51.9, and 57.9 pg/ml in Adjuvant-vaccinated control group (*n* = 5) and 60.3, 62.3, and 68.5 pg/ml in r*Bm*SP44 vaccinated group (*n* = 5), respectively. Results are presented as the mean ± SEM. The data represent two individual experiments. All the levels of cytokine in sera were measured by ELISA kits.

In order to analyze the levels of Th1 and Th2 cytokines in infected mouse sera, the expressions of IFN-γ, TNF-α, and IL-10 were determined by ELISA kits. Compared with the control groups, serum IFN-γ, and IL-10 expression were significantly higher in mice immunized with r*Bm*SP44 on day 6 p.i. Serum TNF-α was also slightly higher in the immunized group than the controls on day 6 and 9 p.i., but the differences were not significant. These data suggested that a Th1/Th2-mixed immune response was induced in r*Bm*SP44 immunized mice during the early stage of infection. This Th1/Th2-mixed immune response may contribute to the protective efficacy of r*Bm*SP44 against *Babesia* parasites infection (Figures 7A–C).

## Discussion

*B. microti*, a major etiological agent of tick-borne babesiosis invading only erythrocytes in human has been sequenced to have the smallest nuclear genome among all apicomplexan parasites (24). Several novel antigens of the parasite were characterized using protein microarrays (25–27). Some of them can trigger host immune response and be associated with genes encoding the secretome and the surface proteome of the parasite (28–30). These studies provide candidates for the development of improved diagnostic assays and vaccines.

A panel of secreted proteins containing signal peptides were identified in previous studies (12, 22), some of which have been characterized as diagnostic antigens of babesiosis. Relative fewer were considered as candidates in vaccine development (31–33). What's more, increased drug resistance of *Babesia* and modest effect of new vaccine candidates were reported (34, 35). In this study, the gene encoding *Bm*SP44 was cloned and characterized in *B. microti*. Bioinformatics analysis suggested that *Bm*SP44 has no homologous genes in other species of *Babesia*; in addition, *Bm*SP44 does not contain any conserved domains, making it difficult to predict its function. *Bm*SP44 is a secreted protein that can be recognized by the host's immune system and therefore considered as an attractive vaccine candidate and a diagnostic marker. Our microarray and ELISA data all indicated that r*Bm*SP44 could be an antigen for serological diagnostics marker for human babesiosis.

As *Bm*SP44 elicits strong immunoreactions, we extended the study to test the role of r*Bm*SP44 in altering *Babesia* infection. In our active immunization experiments, the mice immunized with r*Bm*SP44 demonstrated significant protection against *Babesia* infection. The passive immunization with r*Bm*SP44 rabbit antiserum also delivered protection in immunized mice; however the protection rates were lower than that of active immunization experiments. Although rabbit antibodies have the advantages of recognizing small epitopes (36, 37), the antibody titer of passive immunization was lower than that of active immunization in our study. Also, active immunization may result in cellular immunity in addition to humoral immunity. This may explain why the passive immunization inducing relatively weak protection compared to the active immunization.

As apicomplexan protozoa, the protective immunity induced by *Babesia* proteins like *Bm*MetAP1, *Bm*HSP70 involves mainly cell-mediated responses with high level of IgG1 and Th1 cytokines, such as IFN-γ and IL-12 (38, 39). The interaction between the erythrocyte receptors and MSP1 was critical for the invasion process of *Plasmodium*, another important intraerythrocytic protozoon. Thus, MSP1 is a major malaria vaccine candidate which protects malaria parasites in mouse models (40). Humoral immune responses may also contribute to the protections in these parasite infections. Classically, the Th1 cytokines responses are characterized by the production of IFN-γ, IL-2, and TNF-α, while Th2 responses are represented by cytokine IL-4, IL-6, and IL-10. It was confirmed that cytokines such as IL-12, TNF-α, IFN-γ, and IL-2 play an important role in controlling the proliferation of *Babesia* in the early and acute stages of infection, while IL-10, IL-4, IL-5, and IL-6 may involve in chronic and low parasitemia stages of infections (41–45). Our current study suggested that immunization with r*Bm*SP44 elicits IFN-γ, TNF-α, and IL-10 expression and results in a Th1/Th2 mixed humoral and cellular immune response, which may contribute to protect mice from *Babesia* infection.

The clinical manifestations of *Babesia* infections are diverse and the declining concentration of hemoglobin induced by babesiosis is considered to be a major feature of the infection (42). In our current study, the concentration of hemoglobin in the immunization group was slightly higher than that in controls, while changes in weight and temperature were not discernible. We only observed the condition within 2 weeks after infection, and the increment of temperature is not only associated directly to the concentration of parasites but also related to the immune status of the hosts.

In conclusion, our study indicated that *Bm*SP44 is a secreted protein and localized principally in the cytoplasm of the parasites. *Bm*SP44 can elicit immune responses in a mouse model of Babesiosis and can be recognized by immune serum from different stages of infection. Both active and passive immunization with r*Bm*SP44 (or antisera) can afford protection to mice against *Babesia* infection. Thus, *Bm*SP44 can be used as both a diagnosis marker and a vaccine candidate to combat Babesiosis.

## Data Availability Statement

The datasets generated for this study can be found in the Protein ID: CCF73510.

## Ethics Statement

All animal procedures were conducted in compliance with the principle for the Care and Use of Medical Laboratory Animals (Ministry of Health, People's Republic of China) and approved by the Institutional Animal Care and Use Committee (IACUC) of Soochow University for the use of laboratory animals (Permit Number: ECSU-201800091).

## Author Contributions

XZ and JD conceived the study, collected and analyzed the data, and drafted the manuscript. HW, YW, and JH carried out the whole experiments and revised the manuscript. BX and JH conceived the project and provided technical support for data collection and analysis. All authors read and approved the final manuscript. Written consent to publish was obtained.

## Conflict of Interest

The authors declare that the research was conducted in the absence of any commercial or financial relationships that could be construed as a potential conflict of interest.

## Supplementary Material

Supplementary File 1 The amino acid sequences of *Bm*SP44.

Figure S1 Predicated signal peptide and antigenic epitopes of *Bm*SP44 **(A)** Signal peptide was detected in *Bm*SP44 based on SignalP4.1 software; **(B)** Antigenic epitopes of *Bm*SP44 were predicted by ABCPred bioinformatic serves.

Figure S2 Quantification of the antibody titers in sera of rabbits immunized with r*Bm*SP44. Rabbits immunized with 100 μg **(A)** or 500 μg **(B)** r*Bm*SP44, respectively and the antibody tiers were examined by ELISA. Normal rabbit serum was served as a control.

## References

[1] Vannier E Krause PJ. Human babesiosis. N Engl J Med. (2012) 366:2397–407. 10.1056/NEJMra1202018 22716978

[2] Zhou X Xia S Huang JL Tambo E Zhuge HX Zhou XN. Human babesiosis, an emerging tick-borne disease in the People's Republic of China. Parasite Vectors. (2014) 7:509. 10.1186/PREACCEPT-1503099832120211 25403908 PMC4254216

[3] Vannier E Krause PJ. Babesiosis in China, an emerging threat. Lancet Infect Dis. (2015) 15:137–9. 10.1016/S1473-30991471062-X 25539585

[4] Asensi V González LM Fernández-Suárez J Sevilla E Navascués RÁ Suárez ML. A fatal case of *Babesia divergens* infection in Northwestern Spain. Ticks Tick Borne Dis. (2018) 9:730–4. 10.1016/j.ttbdis.2018.02.018 29496491

[5] Chen Z Li H Gao X Bian A Yan H Kong D. Human Babesiosis in China: a systematic review. Parasitol Res. (2019) 118:1103–12. 10.1007/s00436-019-06250-9 30770979

[6] Wormser GP Prasad A Neuhaus E Joshi S Nowakowski J Nelson J. Emergence of resistance to azithromycin-atovaquone in immunocompromised patients with *Babesia microti* infection. Clin Infect Dis. (2010) 50:381–6. 10.1086/649859 20047477

[7] Krause PJ Human babesiosis. Int J Parasitol. (2019) 49:165–74. 10.1016/j.ijpara.2018.11.007 30690090

[8] Morahan BJ Wang L Coppel RL. No TRAP, no invasion. Trends Parasitol. (2009) 25:77–84. 10.1016/j.pt.2008.11.004 19101208

[9] Moreau E Bonsergent C Al Dybiat I Gonzalez LM Lobo CA Montero E. *Babesia divergens* apical membrane antigen-1 (*Bd*AMA-1): a poorly polymorphic protein that induces a weak and late immune response. Exp Parasitol. (2015) 155:40–5. 10.1016/j.exppara.2015.04.024 25956948

[10] Ord RL Rodriguez M Cursino-Santos JR Hong H Singh M Gray J. Identification and characterization of the rhoptry neck protein 2 in *Babesia divergens* and *B. microti*. Infect Immun. (2016) 84:1574–84. 10.1128/IAI.00107-16 26953328 PMC4862700

[11] Wang G Efstratiou A Adjou Moumouni PF Liu M Jirapattharasate C Guo H. Expression of truncated *Babesia microti* apical membrane protein 1 and rhoptry neck protein 2 and evaluation of their protective efficacy. Exp Parasitol. (2017) 172:5–11. 10.1016/j.exppara.2016.11.001 27876473

[12] Xu B Liu XF Cai YC Huang JL Zhang RX Chen JH. Screening for biomarkers reflecting the progression of *Babesia microti* infection. Parasite Vectors. (2018) 11:379. 10.1186/s13071-018-2951-0 29970143 PMC6029176

[13] Nathaly Wieser S Schnittger L Florin-Christensen M Delbecq S Schetters T. Vaccination against babesiosis using recombinant GPI-anchored proteins. Int J Parasitol. (2019) 49:175–81. 10.1016/j.ijpara.2018.12.002 30684517

[14] Nyalwidhe J Maier UG Lingelbach K. Intracellular parasitism: cell biological adaptations of parasitic protozoa to a life inside cells. Zoology. (2003) 106:341–8. 10.1078/0944-2006-00127 16351918

[15] Piña-Vázquez C Reyes-López M Ortíz-Estrada G de la Garza M Serrano-Luna J. Host-parasite interaction: parasite-derived and -induced proteases that degrade human extracellular matrix. J Parasitol Res. (2012) 2012:748206. 10.1155/2012/748206 22792442 PMC3390111

[16] Lobo CA Rodriguez M Cursino-Santos JR. *Babesia* and red cell invasion. Curr Opin Hematol. (2012) 19:170–5. 10.1097/MOH.0b013e328352245a 22488304

[17] Parker ML Penarete-Vargas DM Hamilton PT Guérin A Dubey JP Perlman SJ. Dissecting the interface between apicomplexan parasite and host cell: Insights from a divergent AMA-RON2 pair. Proc Natl Acad Sci USA. (2016) 113:398–403. 10.1073/pnas.1515898113 26712012 PMC4720339

[18] Frolich S Entzeroth R Wallach M. Comparison of protective immune responses to apicomplexan parasites. J Parasitol Res. (2012) 2012:852591. 10.1155/2012/852591 21876783 PMC3159010

[19] Man S Fu Y Guan Y Feng M Qiao K Li X. Evaluation of a major surface antigen of *Babesia microti* merozoites as a vaccine candidate against *Babesia* infection. Front Microbiol. (2017) 8:2545. 10.3389/fmicb.2017.02545 29312230 PMC5742146

[20] Rodríguez-Galán A Salman AM Bowyer G Collins KA Longley RJ Brod F. An *in vitro* assay to measure antibody-mediated inhibition of *P. berghei* sporozoite invasion against *P falciparum* antigens. Sci Rep. (2017) 7:17011. 10.1038/s41598-017-17274-5 29209029 PMC5717233

[21] Hidalgo-Ruiz M Suarez CE Mercado-Uriostegui MA Hernandez-Ortiz R Ramos JA Galindo-Velasco E. *Babesi*a *bovis* RON2 contains conserved B-cell epitopes that induce an invasion-blocking humoral immune response in immunized cattle. Parasite Vectors. (2018) 11:575. 10.1186/s13071-018-3164-2 30390674 PMC6215676

[22] Zhou X Huang JL Shen HM Xu B Chen JH Zhou XN. Immunomics analysis of *Babesia microt*i protein markers by high-throughput screening assay. Ticks Tick Borne Dis. (2018) 9:1468–74. 10.1016/j.ttbdis.2018.07.004 30017725

[23] Luo Y Jia H Terkawi MA Goo YK Kawano S Ooka H. Identification and characterization of a novel secreted antigen 1 of *Babesia microti* and evaluation of its potential use in enzyme-linked immunosorbent assay and immunochromatographic test. Parasitol Int. (2011) 60:119–25. 10.1016/j.parint.2010.11.001 21070864

[24] Cornillot E Hadj-Kaddour K Dassouli A Noel B Ranwez V Vacherie B. Sequencing of the smallest Apicomplexan genome from the human pathogen *Babesia microti*. Nucleic Acids Res. (2012) 40:9102–14. 10.1093/nar/gks700 22833609 PMC3467087

[25] Xu X Zhang Y Lin D Zhang J Xu J Liu YM. Serodiagnosis of *Schistosoma japonicum* infection: genome-wide identification of a protein marker, and assessment of its diagnostic validity in a field study in China. Lancet Infect Dis. (2014) 14:489–97. 10.1016/S1473-30991470067-2 24656567

[26] Carmona SJ Nielsen M Schafer-Nielsen C Mucci J Altcheh J Balouz V. Towards high-throughput immunomics for infectious diseases: use of next-generation peptide microarrays for rapid discovery and mapping of antigenic determinants. Mol Cell Proteomics. (2015) 14:1871–84. 10.1074/mcp.M114.045906 25922409 PMC4587317

[27] Cornillot E Dassouli A Pachikara N Lawres L Renard I Francois C. A targeted immunomic approach identifies diagnostic antigens in the human pathogen *Babesia microti*. Transfusion. (2016) 56:2085–99. 10.1111/trf.13640 27184823 PMC5644385

[28] Silva JC Cornillot E McCracken C Usmani-Brown S Dwivedi A Ifeonu OO. Genome-wide diversity and gene expression profiling of *Babesia microti* isolates identify polymorphic genes that mediate host-pathogen interactions. Sci Rep. (2016) 6:35284. 10.1038/srep35284 27752055 PMC5082761

[29] Elton CM Rodriguez M Ben Mamoun C Lobo CA Wright GJ. A library of recombinant *Babesia microti* cell surface and secreted proteins for diagnostics discovery and reverse vaccinology. Int J Parasitol. (2019) 49:115–25. 10.1016/j.ijpara.2018.10.003 30367868 PMC6406021

[30] Magni R Luchini A Liotta L Molestina RE. Analysis of the *Babesia microti* proteome in infected red blood cells by a combination of nanotechnology and mass spectrometry. Int J Parasitol. (2019) 49:139–44. 10.1016/j.ijpara.2018.08.004 30391228 PMC10548858

[31] Jia H Terkawi MA Aboge GO Goo YK Ma L Zhou J. Identification of secreted antigen 3 from *Babesia gibsoni*. Clin Vaccine Immunol. (2009) 16:944–8. 10.1128/CVI.00087-09 19386799 PMC2691051

[32] Goo YK Aboge GO Terkawi MA Jia H Yamagishi J Sunaga F. Four promising antigens, *Bg*P32, *Bg*P45, *Bg*P47, and *Bg*P50, for serodiagnosis of *Babesia gibsoni* infection were classified as B. *gibsoni* merozoite surface protein family. Parasitol Int. (2012) 61:364–8. 10.1016/j.parint.2011.11.007 22172478

[33] Zhan X Yu L An X Liu Q Li M Nie Z. Evaluation of *Babesia gibsoni* GPI-anchored protein 47 (BgGPI47-WH) as a potential diagnostic antigen by enzyme-linked immunosorbent assay. Front Vet Sci. (2019) 6:333. 10.3389/fvets.2019.00333 31681802 PMC6797833

[34] Lubin AS Snydman DR Miller KB. Persistent babesiosis in a stem cell transplant recipient. Leuk Res. (2011) 35:e77–8. 10.1016/j.leukres.2010.11.029 21185598 PMC3085102

[35] Szymczak J Kozłowska J Doligalska M. Evaluation of inhibitory effect of redox-active antimalarial drug against *Babesia microti* in mice. Ann Parasitol. (2017) 63:223–7. 10.17420/ap6303.109 29274217

[36] Vilches-Moure JG Ramos-Vara JA. Comparison of rabbit monoclonal and mouse monoclonal antibodies in immunohistochemistry in canine tissues. J Vet Diagn Invest. (2005) 17:346–50. 10.1177/104063870501700407 16130992

[37] Seeber S Ros F Thorey I Tiefenthaler G Kaluza K Lifke V. A robust high throughput platform to generate functional recombinant monoclonal antibodies using rabbit B cells from peripheral blood. PLoS ONE. (2014) 9:e86184. 10.1371/journal.pone.0086184 24503933 PMC3913575

[38] Terkawi MA Aboge G Jia H Goo H Ooka YK Yamagishi J. Molecular and immunological characterization of *Babesia gibsoni* and *Babesia microti* heat shock protein-70. Parasite Immunol. (2009) 31:328–40. 10.1111/j.1365-3024.2009.01109.x 19493212

[39] Munkhjargal T Aboge GO Ueno A Aboulaila M Yokoyama N Igarashi I. Identification and characterization of profilin antigen among *Babesia* species as a common vaccine candidate against babesiosis. Exp Parasitol. (2016) 166:29–36. 10.1016/j.exppara.2016.03.024 27003460

[40] Renia L Goh YS. Malaria Parasites: The Great Escape. Front Immunol. (2016) 7:463. 10.3389/fimmu.2016.00463 27872623 PMC5098170

[41] Chen D Copeman DB Burnell J Hutchinson GW. Helper T cell and antibody responses to infection of CBA mice with *Babesia microti*. Parasite Immunol. (2000) 22:81–8. 10.1046/j.1365-3024.2000.00279.x 10652120

[42] Brown AL Shiel RE Irwin PJ. Clinical, haematological, cytokine and acute phase protein changes during experimental *Babesia gibsoni* infection of beagle puppies. Exp Parasitol. (2015) 157:185–96. 10.1016/j.exppara.2015.08.002 26297954

[43] Roussilhon C Bang G Bastaert F Solhonne B Garcia-Verdugo I Peronet R. The antimicrobial molecule trappin-2/elafin has anti-parasitic properties and is protective *in vivo* in a murine model of cerebral malaria. Sci Rep. (2017) 7:42243. 10.1038/srep42243 28181563 PMC5299836

[44] Djokic V Akoolo L Parveen N. *Babesia microti* infection changes host spleen architecture and is cleared by a Th1 immune response. Front Microbiol. (2018) 9:85. 10.3389/fmicb.2018.00085 29445365 PMC5797759

[45] Zhou X Wang H Xue JB Xia S Zhou XN. [Epidemic and research progress of babesiosis]. Zhongguo Xue Xi Chong Bing Fang Zhi Za Zhi. (2019) 31:63–70. 10.16250/j.32.1374.2018293 31016926

