# Supplementary material for: Babesia microti Protein BmSP44 Is a Novel Protective Antigen in a Mouse Model of Babesiosis
Source: Front Immunol. 2020 Jul 7;11:1437. doi: 10.3389/fimmu.2020.01437 (PMC7358449; doi:10.3389/fimmu.2020.01437)
Supplement: Supplementary File 1 — The amino acid sequences of BmSP44. [file Table_1.DOCX]

**Amino acid sequences of *Bm*SP44** (XP_012648119)**:**

MHINYKLIITGLVSIALATSITLAVIYLPNRSCPGNNGVGGGSGDNNSGIIPNDPHPCCNNLRQKPQYQTKPENELVNDDRDLNFNKIRGGKQIITFTVPSIDDLKNKRLSDSEFILSEKANPLISSGDSKNVIVFEVKNDNEKLMGSVEVGQWEVTITTSCIRRIVIFDSNEVSDNIPMYIYIVDYFEGGNSTVSKFFFANNRWNADFTNHTPNAA
