# Supplementary figures and images for: Babesia microti Protein BmSP44 Is a Novel Protective Antigen in a Mouse Model of Babesiosis
Source: Front Immunol. 2020 Jul 7;11:1437. doi: 10.3389/fimmu.2020.01437 (PMC7358449; doi:10.3389/fimmu.2020.01437)

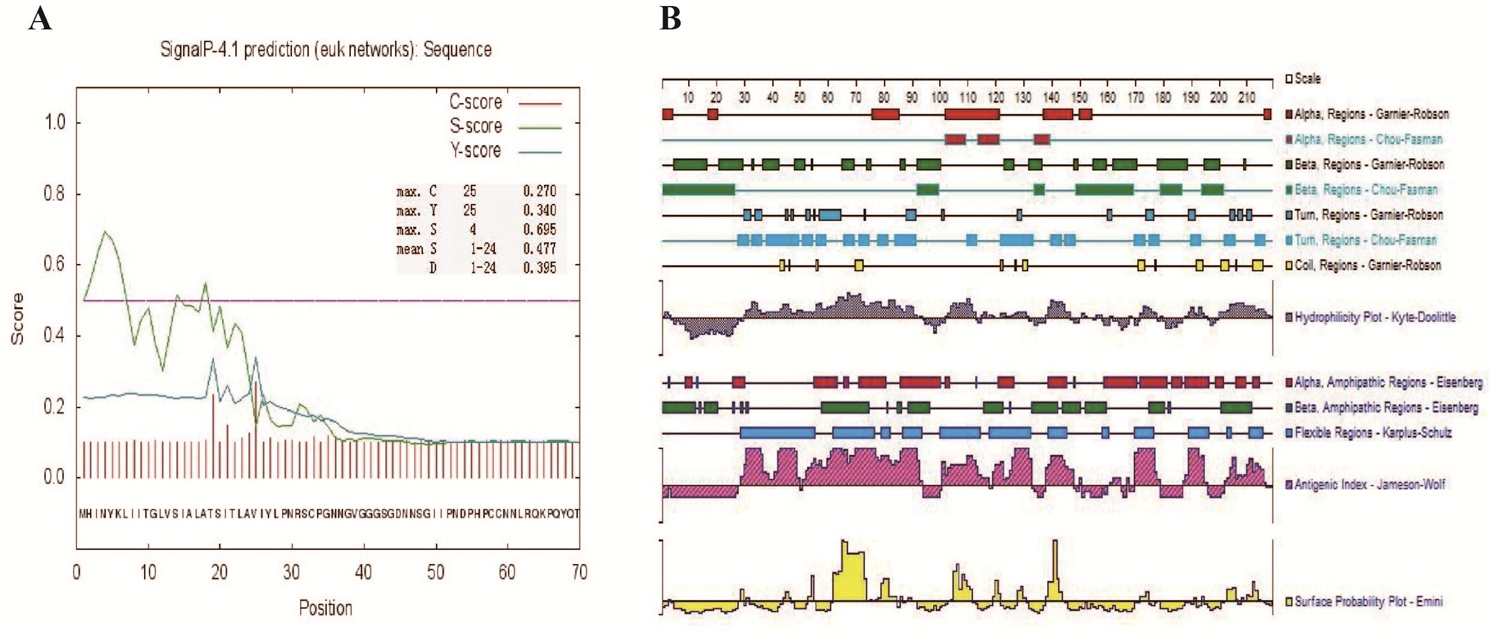

Supplement: Figure S1 — Predicated signal peptide and antigenic epitopes of BmSP44 (A) Signal peptide was detected in BmSP44 based on SignalP4.1 software; (B) Antigenic epitopes of BmSP44 were predicted by ABCPred bioinformatic serves. [file Image_1.JPEG]

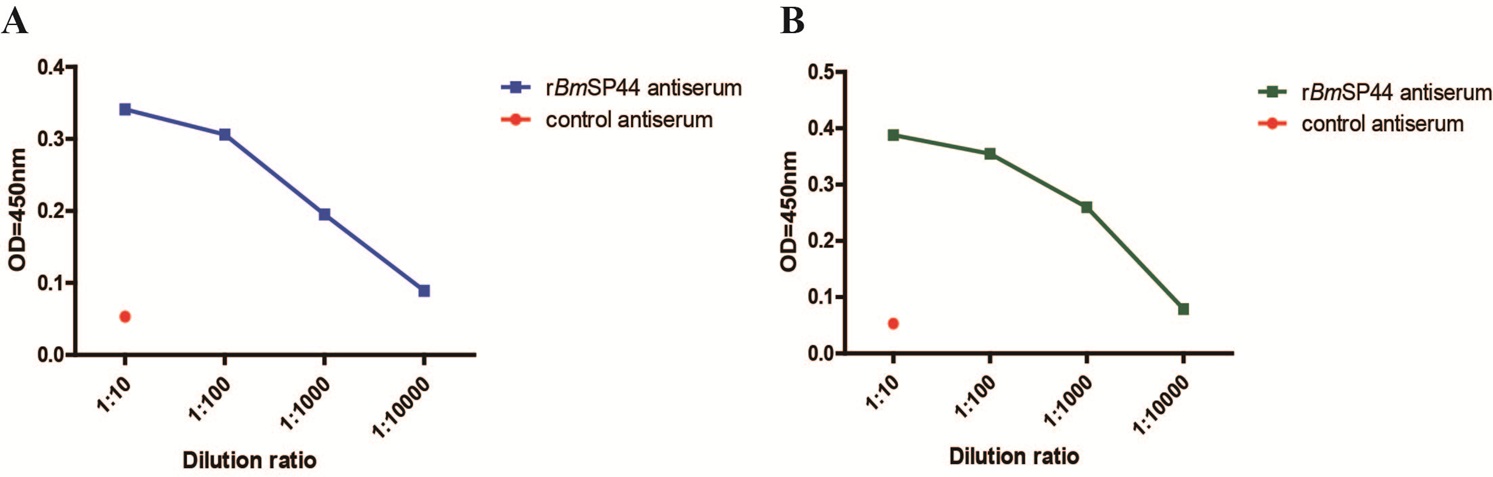

Supplement: Figure S2 — Quantification of the antibody titers in sera of rabbits immunized with rBmSP44. Rabbits immunized with 100 μg (A) or 500 μg (B) rBmSP44, respectively and the antibody tiers were examined by ELISA. Normal rabbit serum was served as a control. [file Image_2.JPEG]
